# Social media features, perceived group norms, and adolescents’ active social media use matter for perceived friendship quality

**DOI:** 10.3389/fpsyg.2024.1222907

**Published:** 2024-04-24

**Authors:** Federica Angelini, Gianluca Gini, Claudia Marino, Regina Van Den Eijnden

**Affiliations:** ^1^Department of Developmental and Social Psychology, University of Padua, Padua, Italy; ^2^Department of Interdisciplinary Social Science, Youth Studies, Utrecht University, Utrecht, Netherlands

**Keywords:** social media features, active social media use, perceived norms, friendship quality, adolescence

## Abstract

**Introduction:**

Developmental researchers are becoming increasingly interested in the role of social media in adolescents’ experiences with their peers; however, to date, few studies have investigated the association between youngsters’ interactions with friends online and the perceived quality of their friendship relationships.

**Methods:**

Thus, the current study aims to test the associations between three social media features, as perceived by adolescents, (i.e., availability, quantifiability, visualness) and the quality of adolescents’ friendships (in terms of perceived validation, intimacy, companionship), considering participants’ frequency of active social media use (i.e., self-oriented and other-oriented social media use), and the role of perceived group norms about social media use. Moreover, we tested potential differences across gender groups. Participants were 751 adolescents (*M*_age_ = 16.2, SD = 1.5).

**Results:**

A SEM analysis showed that, among the perceived social media features, availability was positively associated with perceived friendship quality–both directly and indirectly. Furthermore, friends who participated more in other-oriented social media use reported being more satisfied with their friendship relationships and the results showed that peer influence processes were also active on social media.

**Discussion:**

Taken together, these results emphasize the study of social media as a social context for a better understanding of contemporary peer experiences during adolescence. Specifically, novel behaviors (e.g., liking or commenting on posts or content of peers), which characterize interactions between friends, may support relational functioning and well-being purposes in both the offline and online context.

## 1 Introduction

The intrinsic interactive nature of social media platforms makes them particularly appealing for contemporary adolescents, who engage in daily online activities to get in touch and communicate with peers (Moreno and Uhls, 2019). Social relationships are indeed pivotal during adolescence, when interactions with friends become more frequent, relevant, and intimate. Over the last decade, developmental researchers have been increasingly interested in comprehending the significance of social media use on youth’s interactions (e.g., Uhls et al., 2017), particularly within the realm of relationships with friends (e.g., Pouwels et al., 2021). In this regard, Nesi et al. (2018a,b) have recently outlined the *Transformation Framework*, which provides a useful theoretical context for studying peer relationships on social media. Compared to former times, adolescents’ social media use is indeed characterized by new forms of interaction with the peer (friend) network, which include much more immediacy and visual communication (e.g., posting or commenting and liking on videos and photographs) and that can change or compensate interactions experienced offline (Nesi et al., 2018a,b; Maheux et al., 2020). Moreover, to better capture the complexity of social media use, the distinction between nuanced online activities (i.e., self-oriented and other-oriented social media use), which could be relevant to relational well-being, is increasingly considered (Nesi et al., 2021). Further, the strong effect of adherence to peer group norms in influencing adolescents’ behavior, broadly confirmed by “offline” developmental research (Prinstein and Dodge, 2008), is now also considered on social media, where is equally present since adolescents are more likely to engage in online behaviors that are perceived as frequent and valued among their friends (Brechwald and Prinstein, 2011; Marino et al., 2020). However, there is a lack of research on how the way adolescents perceive the social media context and the way they and their friends communicate and behave online could be associated with the quality of their friendship relationships. To contribute to filling this gap, the current study aims to investigate possible associations between perceived context of social media and adolescents’ perceived quality of their friendship relations. In examining these associations, we take into consideration the role of participants’ frequency of active social media use (i.e., self-oriented and other-oriented social media use), the value they attribute to it, and the perceived frequency of their friends’ other-oriented use (in this study conceptualized in terms of perceived peer group norms).

### 1.1 Friendship quality in the social media context

Adolescents’ relationships with friends are characterized by spending time together and having fun, sharing intimate information, and being supportive to each other (Bukowski et al., 2009). Contrary to previous generations, online spaces represent an additional important means of social connection with friends for today’s youth (Moreno and Uhls, 2019) in a way that social media can play a predominantly beneficial role in supporting offline friendship relationships during pre-adolescence and adolescence (Angelini et al., 2022; Meeus et al., 2022). Although the social media context may be somewhat different from the traditional offline context, a recent study has shown that the dynamics underlying the perceived quality of friendship do not vary significantly from offline to online interactions (Yau and Reich, 2018). Indeed, just like when in person, adolescents on social media are able to show affection, get more intimate and have fun with friends, simply in new ways (e.g., by sharing private photos or posting something to reciprocally experience feelings of being special and important). Specifically, Yau and Reich (2018) suggested that the core components that contribute to perceived friendship quality within the offline context, as defined by Parker and Asher (1993) (i.e., validation, intimacy, instrumental support, companionship, conflict, and conflict resolution) would not differ between offline and online interactions. In this study, we focused on three dimensions of friendship quality, namely (i) validation (caring for a friend and making them feel special by showing support and interest), (ii) intimacy (sharing personal thoughts and feelings with another), and (iii) companionship (dedicating time to having fun with a friend and relaxing together). These dimensions were selected as key components of friendship quality because they are the most relevant during adolescence and best representative of the definition of close friendship itself (Bukowski et al., 2009).

### 1.2 The social context of social media

This study was structured to align with the core principles of the above-mentioned Transformation Framework (Nesi et al., 2018a,b, 2021). According to this recent integrative theoretical model, social media represent a “real” social context, only partially similar to the traditional offline one, and characterized by eight features (i.e., asynchronicity, permanence, availability, publicness, cue absence, quantifiability, visualness, and algorithm) which may contribute to *transform* adolescents’ behaviors and experiences, including friendship relationships (Nesi et al., 2018a,b). For instance, social media can allow for more frequent, immediate, and qualitatively different peer interactions, leading to easier and faster connections with friends and a greater opportunity to receive social support. At the same time, this can also amplify expectations and requests within friendships, such as expectations of being constantly present and available online and requests to be quickly responsive. Furthermore, social media features can make certain behavior easier when implemented online, for example, providing marginalized teens with more opportunities to connect with peers (Angelini and Gini, 2023). Finally, social media have introduced unique experiences, not possible in the traditional offline context, such as managing and enhancing one’s online presentation (Nesi et al., 2018a,b). To address the aims of the present study, we took into consideration three main characteristics of social media that, in a previous exploratory study (Angelini et al., 2022), were found to be positively linked with the key components of friendship quality; specifically, we focused on (i) availability (i.e., the ease with which content can be shared on social media, regardless of physical location), (ii) quantifiability (i.e., the allowance for countable social metrics, such as likes or number of followers), and (iii) visualness (i.e., the extent to which photographs and videos are emphasized by a given social media platform) (Nesi et al., 2018a). In a sample of Italian adolescents, in fact, we found preliminary, but important confirmation for the hypothesized role of adolescents’ perceived social media functioning in sustaining their friendship relations (Angelini et al., 2022). More in detail, validation was found to be directly associated with both visualness and quantifiability. In other words, communicating and expressing oneself by posting photos and videos (i.e., visualness) and relying on social indicators such as comments or “likes” (i.e., quantifiability) might contribute to validate the relationship with friends and by fostering feelings of being special and important to each other. Moreover, the more adolescents perceived availability as a key feature of social media, the greater they experienced companionship with friends. Further, perceived availability and visualness of social media were found to be associated with a higher perceived online social support and with a stronger tendency to express emotions online, which, in turn, were associated with higher levels of experienced intimacy and validation in the relationship with friends (Angelini et al., 2022). Although there is still scant research that analyzes adolescents’ social media interaction with friends in association with the quality of their friendship experiences, and even less is known about the role of specific characteristics of social media, these results are consistent with the evidence that friendship closeness mostly relies on social and emotional support from peers (e.g., Rousseau et al., 2019; Batool and Lewis, 2020) and that social media may play a role in this association by supporting youth’s friendship formation and maintenance (Amichai-Hamburger et al., 2013; Uhls et al., 2017). For example, in contrast with the offline context, adolescents’ needs to stay constantly in touch with friends can be now rapidly fulfilled thank to availability of social media; moreover, many functionalities of social media (i.e., visual communication) allow adolescents to experience new forms of expression (i.e., sharing feelings and thoughts through visual content) that contribute to their self-presentation and identity development (Valkenburg et al., 2016). Similarly, unique social media features make these platforms particularly appealing for young individuals since they allow to satisfy specific developmental needs during adolescence (Orben, 2020), such as making experiences of immediate social feedback or emotional support from friends with one click (e.g., through likes, or positive comments on their social media content, that is quantifiability) (Yau and Reich, 2018; Nesi et al., 2018a,b; Pouwels et al., 2021).

Thus, based on previous research and with the aim of overcoming the exploratory design of our previous study (Angelini et al., 2022), we decided to focus on the three mentioned social media characteristics (i.e., availability, quantifiability, and visualness) as key dimensions of social media context in association with friendship quality.

However, to date, little is known about the association between adolescents’ perception of specific characteristics of the social media context and the online activities they and their friends engage in (e.g., chatting, posting content, “liking” or commenting on friends’ posts). Even though social media are likely to present each feature to a certain degree, each of them can be expected to play a unique role in adolescents’ and their friends’ behavior on social media and, ultimately, in explaining friendship quality. In the next paragraph, we discuss the way contextual functioning of social media (in terms of perceived availability, quantifiability, and visualness) may stimulate adolescents’ engagement in specific social media activities with friends and, as a result, be associated with a higher perception of friendship quality.

### 1.3 Adolescents active social media use

Social media allow users, adolescents in particular, to create and exchange their own-generated content (Kaplan and Haenlein, 2010) in the form of photos, videos, or status updates, and to interact with other users’ content, for example by “liking” and commenting on them. At the same time, social media users can simply browse through others’ profiles, without any interaction being required (Rousseau et al., 2017). Thus, the complexity of social media activities can be classified into two categories: “passive” and “active” social media use (Verduyn et al., 2017). Passive social media use, on the one hand, refers to the mere exposure to other people’s online lives (e.g., by scrolling through their profiles without interacting with them), and does not involve the creation of any content to be shared. Active social media use, on the other hand, is based on posting personal content that can receive feedback (e.g., likes and comments), and on direct exchanges and interaction with the online network (e.g., by chatting, sharing photos, or status updates) (Verduyn et al., 2017; Escobar-Viera et al., 2018; Thorisdottir et al., 2019; Steinsbekk et al., 2021). Research on active and passive use of social media has discussed different outcomes on adolescents’ well-being and emotional health; passive use, for example, has been associated with depression and lower well-being and has been described as a risk factor for social comparison and body dissatisfaction; conversely, active use appears to be associated with higher well-being and increased social and emotional support from friends, thus improving both self-esteem and peer relations (Escobar-Viera et al., 2018; Orben, 2020).

Given the noninteractive nature of passive social media use, beyond interaction between the user and the platform itself, and the assumed positive effects of active social media use on social relationships, the current study focuses on the role of active social media use. Indeed, such use could be more clearly associated with enhanced friendship quality. Specifically, we differentiated between the two dimensions of active use proposed by Steinsbekk et al. (2021), namely self-oriented and other-oriented social media use. Self-oriented social media use (henceforth “self-oriented use”) refers to activities that involve posting original content, updates, and photos on one’s own page, whereas other-oriented social media use (henceforth “other-oriented use”) consists in interacting with and reacting to others’ content, by liking or commenting on others’ posted material. Although previous studies on online peer experiences and friendship quality focused mainly on general social media use (e.g., Pouwels et al., 2021), without clearly distinguishing between more nuanced activities, we believe that active social media use, especially other-oriented use, may better account for the role that social media use play in adolescents’ perceived quality of friendship relationships.

Furthermore, we were not only interested in considering the frequency of adolescents’ active social media use, but we also took into consideration the importance they give to active, other-oriented social media use (henceforth “beliefs on other-oriented use”), as a valuable activity when interacting with friends online (e.g., “I think it is important to ‘like’ or comment on my friends’ posts”). In other words, we hypothesized that stronger (i.e., positive) beliefs on other-oriented use would be associated with more frequent active social media use, especially other-oriented use, that, in turn, would be associated with a higher perceived validation, companionship and intimacy within friendship relationships.

Regarding the three social media features described above, we expected that all of them could stimulate adolescents’ participation in active social media use and foster their beliefs about it and, in turn, be associated with perceived quality of friendships. For example, the fact that social media are characterized by a high degree of availability of content posted by others may increase the likelihood that adolescents engage in other-oriented activities, such as liking or commenting on friends’ posts to show appreciation and support as soon as the content is posted; this kind of activity is also allowed by the possibility to give social feedback on social media (i.e., quantifiability), compared to offline interactions. Similarly, the easy access to social media platforms may stimulate the activity to post new content with the aim of receiving friends’ feedback in terms of like and comments, which foster social reward mechanisms (Nesi and Prinstein, 2018). Most social media, indeed, offer adolescents the possibility to literally count social information, in a way that would not be possible offline, and this can be used as an important indicator for adolescents’ online self-presentation and evidence of one’s social status (at least online). In this regard, the perceived emphasis on visual communication through photos and videos on social media (i.e., visualness) may be associated with the frequency of adolescents’ use of social media both to share new visual content and to interact with the visual content posted by their friends. For example, some platforms more than others (e.g., Instagram or TikTok), place a great emphasis on visual content, particularly when they are appealing or catchy; as in the case of quantifiability, adolescents may engage in active social media use and share visually-oriented posts so that they are more likely to be seen and liked by peers. Therefore, we believe that unique features of social context are strictly related to the choice of specific activities on social media, which could be associated with greater satisfaction in friendship.

### 1.4 Perceived group norms about other-oriented use

Beyond social media features, another “contextual” dimension that may play an important role in adolescents’ online behavior and their perception of friendship quality refers to their friends’ use of social media, as perceived by participants (i.e., perceived group norms about friends’ online activity). Developmental research has consistently confirmed the strong effect of peer influence on adolescents’ attitudes and behaviors, through different mechanisms (e.g., adherence to peer group norms, homophily, social identity, etc.; Cook et al., 2007; Prinstein and Dodge, 2008). According to the Social Influence Theory (Kelman, 1974), adolescents engage in activities that are considered valuable by the group members and, both offline and online, they are more likely to behave and think as the majority of their peers do (Brechwald and Prinstein, 2011). Furthermore, according to the Transformation Framework, key characteristics of the online environment may intensify traditional experiences, such as peer influence processes (Nesi et al., 2018a,b). Regarding the relations between peer influence processes and adolescents’ online behavior, previous studies have mainly focused on the role of social norms on online risky behaviors, such as problematic social media use (e.g., Marino et al., 2020) or internet gaming disorder (e.g., Haagsma et al., 2013). However, little is known about whether perceived group norms may play a role in adolescents’ daily, non-problematic social media use. Moreover, to our knowledge, no studies have explored the concurrent contribution of social media context (i.e., social media features) and adolescents’ social context (i.e., peer group norms) to perceived friendship quality. In the present work, social norms of social media use are conceptualized in terms of friends’ use, which was operationalized as adolescents’ perception of the frequency with which their friends use social media in an other-oriented way (paralleling participants’ evaluation of their own other-oriented use of social media). Consistent with the literature on between-friends similarity (e.g., Mazur and Richards, 2011; Marino et al., 2020), we expected that higher (perceived) frequency of other-oriented use by friends would be associated with higher frequency of other-oriented use by participants, as well as to their beliefs about it, and, in turn, with their perceived friendship quality.

### 1.5 Gender differences in friendship quality and social media use

To our knowledge, little research exists about gender differences in adolescents’ social media use in association with friendship quality. For example, Pouwels et al. (2021) recently found a gender difference in the association of specific social media use (i.e., WhatsApp) with friendship closeness, which was found to be negative only for male adolescents. Similarly, in a previous study (Angelini et al., 2022)we found that males and females do differ in the way they perceive social media features and their associations with friendship quality.

Regarding perceived friendship quality, it is well known that females tend to create more intimate and self-disclosed relationships. Moreover, they generally report having higher expectations for companionship and validation within close friendships. Males, on the other hand, seem to rely more on shared activities with friends to feel satisfied with their relations (Parker and Asher, 1993; Yau and Reich, 2018). Concerning existing gender differences in social media use, we know that female adolescents show higher levels of engagement in social media and tend to be more active online than males. Specifically, to express themselves, females seem to post more frequently and to rely more on visual communication than males (e.g., by sharing photos and videos) (Nesi and Prinstein, 2018; Prinstein et al., 2020).

Furthermore, results from our previous study (Angelini et al., 2022) showed that females, compared to males, reported feeling more validated within friendship relations when they have increased online access to friends (i.e., availability of social media), indicating a tendency toward preferring online conversations. Males, instead, showed to rely more on countable metrics (e.g., “likes”) to validate the relationships with friends and, as for the offline context, they tend to share less their emotions with friends on social media.

Based on previous findings, we may expect the association of social media features with friendship quality via active social media use and perceived group norms about social media use to differ across gender groups. However, given the lack of existing research specifically focused on these variables, specific *a priori* hypotheses were not formulated for this study and the analyses were considered exploratory.

### 1.6 The current study

Building on the theoretical tenets of the Transformation Framework (Nesi et al., 2018a,b) and previous studies discussed above (e.g., Yau and Reich, 2018; Angelini et al., 2022), in the current study we were interested in exploring the concurrent contribution of perceived social media context (represented by three social media features, namely availability, quantifiability, and visualness), adolescents’ frequency of active social media use (i.e., self-oriented and other-oriented use), and their beliefs about other-oriented use, to perceived friendship quality (in terms of validation, intimacy, and companionship). In addition, we were interested in examining to what extent perceived group norms about other-oriented use would be associated with participants’ active social media use and with their beliefs about it.

**Figure 1:**
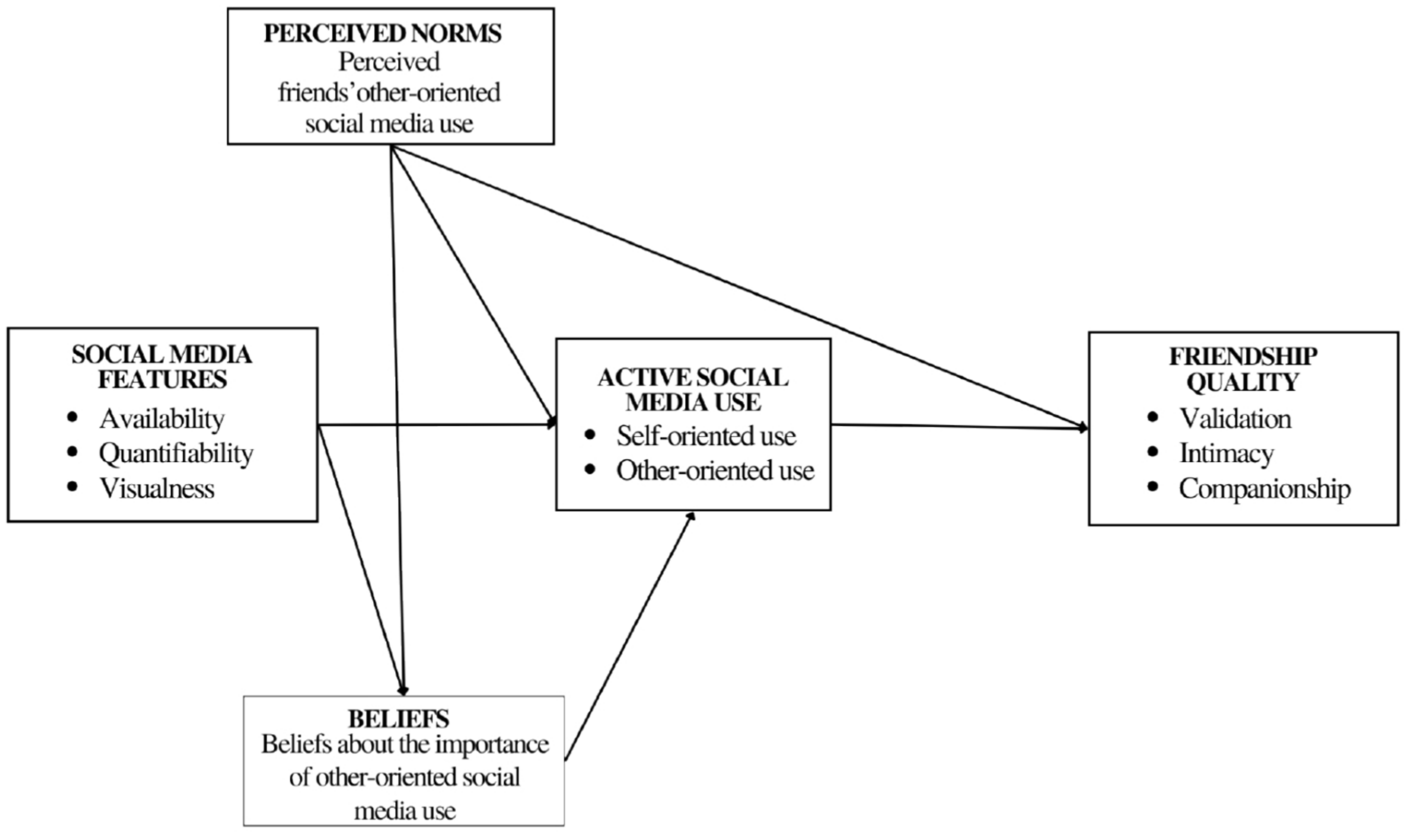
Hypothesized model.

The conceptual model (Figure 1) was designed to test the following hypotheses. First, we hypothesized that adolescents’ friendship quality would be associated with the extent to which adolescents and their friends (as perceived by participants) use social media in an other-oriented way. Specifically, due to the reciprocal nature of friendship relations, we expected a positive direct effect of both participants’ other-oriented use (H1a) and perceived friends’ other-oriented use (H1b) on friendship quality. That is, higher other-oriented use, both by participants and their friends, should be associated with higher friendship quality. Second, with regard to peer influence processes during adolescence, we hypothesized that perceived group norms, would be positively associated with adolescents’ active social media use, especially other-oriented use, both directly (H2a) and indirectly via participants’ beliefs on other-oriented use (H2b). Moreover, as far as the specific role of social media context is concerned, we expected that it would help explain perceived friendship quality via adolescents’ orientation of social media use and their beliefs about it. Specifically, we hypothesized that adolescents’ perception of the different social media features (i.e., availability, quantifiability, and visualness) would be linked to their beliefs on other-oriented use and their type of social media use (self- vs. other-oriented use) (H3a) and, in turn, to perceived friendship quality (H3b). Finally, a further objective of the present study was to examine possible differences in the tested associations between gender groups (males vs. females).

## 2 Materials and methods

### 2.1 Sample and procedure

Participants were adolescents attending grades 9–12th in Italian public high schools. The whole sample included 751 adolescents (56.6% females) aged between 13 and 20 years (*M_age_* = 16.2 years; *SD* = 1.5). All participants indicated that they were engaged with at least one social media platform on a regular basis and 42% of them reported using three or more platforms. The most used social media were WhatsApp, Instagram, and YouTube (respectively, 99.5, 91.1, and 80.8% of the sample), followed by TikTok (74.2%), Snapchat (24.5%), and Facebook (7.1%). These figures align with recent statistics on the use of social media among adolescents (van Driel et al., 2019). One of the last questions in the questionnaire asked participants to report the social media they were referring to during the compilation. Most of adolescents (64.9%) reported having considered social media in general, while the most frequently mentioned social media for those who had thought about a specific one were Instagram (68.3%) and WhatsApp (27%).

The protocol and procedure of the present study were approved by the local Ethics Committee for Psychological Research (protocol no. 4,170). Before participants filled out a set of anonymous self-report questionnaires, permissions from schools were obtained, and parents—or students aged 18 years or over signed active consent. Data were collected during a regular school day between November 2021 and May 2022. Participants accessed the link to the online questionnaire in computer rooms at school or, when required by COVID-19 pandemic regulations, during a Zoom session, always in the presence of a teacher and a research assistant. Confidentiality was ensured and participants were informed they could leave the study at any stage without consequences; finally, the researchers thanked participants for their time and they remained available to clarify any doubts.

The data used in the current study are original and have not been used in previous studies.

### 2.2 Measures

#### 2.2.1 Perceived friendship quality

To measure adolescents’ perceived friendship quality, we used items from the Friendship Quality Questionnaire–Revised (Parker and Asher, 1993; Nangle et al., 2003). The original scale includes a total of 18 items, which cover six dimensions of friendship quality: Validation, intimacy, conflict, conflict resolution, help, and companionship. To note that, in this study, we were not interested in differentiating between specific aspects of friendship, but in explaining perceived friendship quality as a broader concept. Thus, we focused on the following three dimensions as best representation of the definition of friendship quality (Bukowski et al., 2009), specifically: Validation (e.g., “My friends and I make each other feel feeling good about what we think”), Intimacy (e.g., “My friends and I always talk about the things that make us sad”), and Companionship (e.g., “My friends and I think about the things we could do together”). Prior to answer these items we asked participants to think about their friendship relationships (which we defined as “strong and positive emotional relationship, characterized by reciprocity and a sense of closeness that exists between two or more people. Friends are people with whom you spend a lot of time and with whom you share interests, thoughts, and experiences”) and to rate items on a five-point scale (from 1 = not at all true to 5 = completely true).

**Figure 2:**
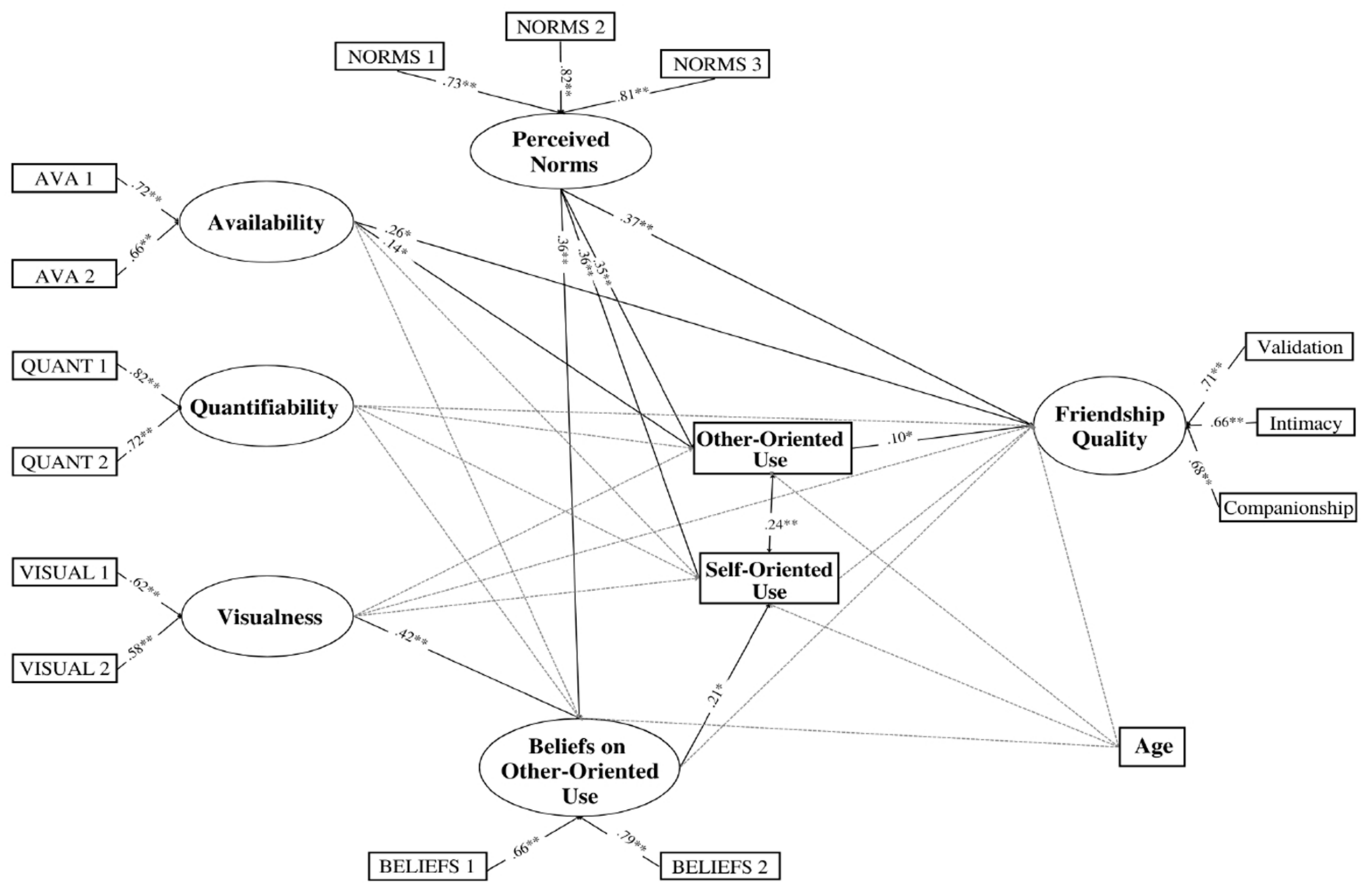
Model of relationship between the study variable in the whole sample. *N =* 751; ^*^p < 0.05, ^**^p < 0.001. For sake of clarity, only significant associations are reported. Full results are available upon request.

In this sample, the scale confirmed a good factorial structure (CFA: χ^2^ = 96.536, *p* < 0.001, CFI = 0.962, RMSEA = 0.063, SRMR = 0.036). The internal consistency was Cronbach’s α = 0.70 (95% CI = 0.66–0.74), McDonald’s ω = 0.71, for validation; α = 0.71 (95% CI = 0.67–0.74), ω = 0.71, for intimacy; α = 0.74 (95% CI = 0.71–0.77), ω = 0.75, for companionship. Answers to each item were averaged for each dimension, then a latent variable for “friendship quality” was computed within the model (factor loadings are reported in Figure 2).

#### 2.2.2 Perceived social media features

For the purpose of the current study, items from the Perceived Social Media Features Scale (Angelini et al., 2022) were used. This scale was developed to contribute filling the gap about the lack of a proper measure for the perceived presence of the social media features, while emphasizing adolescents’ actual perception of how social media are built and work. In addition, this measure allows to disentangle the unique role of each feature, beyond focusing on specific platforms. Specifically, the whole scale includes two items per social media feature (16 items in total), which were developed in Italian starting from their conceptual definition (Nesi et al., 2018a, 2020). In this study, we used items to measure Availability (two items: “I think it is easy to share new content on social media or to access content shared by others regardless of where we are (e.g., even when we are distant from those people),” “I think it is easy to contact other people and communicate with them through social media, even when we are distant”; McDonald’s ω = 0.64), Quantifiability (two items: “I think that on social media it is easy to count the number of friends or followers, and the comments, reactions or sharing of the posts that I have shared,” “I think that on social media it is easy to count the number of friends or followers, and comments, reactions or sharing of posts that others have shared”; ω = 0.74), and Visualness (two items: “I think that on social media, photos and videos allow you to express yourself,” “I think that on social media communication through photos and videos is very important,” ω = 0.53). The scale has already been used with Italian adolescents and has shown good factorial validity (Angelini et al., 2022). In this sample, the scale confirmed a good factorial structure (CFA: *χ*^2^ = 9.472, *p* = 0.149, CFI = 0.996, RMSEA = 0.028, SRMR = 0.016).

Participants rated their level of agreement with each item on a five-point scale (from 1 = not at all, to 5 = completely true). For each feature, a latent variable was then computed with the two respective items (factor loadings are reported in Figure 2).

#### 2.2.3 Active social media use

Participants indicated the characteristics of their use of social media answering two single items adopted and revised by Steinsbekk et al. (2021). In the original version, participants were asked to report their frequency of active social media use per month and to distinguish between (a) posting content in general and (b) posting photographs, and between (a) commenting on and (b) liking others’ posts. In the current study the first item measured the number of time per week participants enact *Self-oriented social media use* (e.g., “How often do you post something (status update, photos, or other content) on your social media in a week?”) while the other one referred to *Other-oriented social media use* (e.g., “How often do you comment or ‘like’ your friends’ posts (status updates, photos, or other content) in a week?”). Answers to each item were given on a five-point scale (from 1 = never to 5 = very often) and two manifest variables were used to indicate self-oriented and other-oriented social media use, respectively. Higher scores indicate higher frequency of self-oriented or other-oriented use, respectively.

#### 2.2.4 Perceived group norms about other-oriented social media use

To assess perceived friends’ use of social media in an other-oriented way three items were created *ad-hoc* for this study (i.e., “My friends react to my posts/stories with likes or comments,” “My friends tag me in a post/story,” and “My friends share photos or videos on social media depicting them and me together”). In this sample, the internal consistency was Cronbach’s α = 0.82 (95% CI = 0.79–0.84), McDonald’s ω = 0.82. Participants reported the perceived frequency of their friends’ other-oriented social media use during a week, rating each item on a five-point scale (from 1 = never to 5 = very often), so that higher scores indicate higher frequency of perceived friends’ other-oriented social media use. A latent variable was computed with the three items (factor loadings are reported in Figure 2).

#### 2.2.5 Beliefs on other-oriented social media use

To assess adolescents’ beliefs about the importance of using social media in an other-oriented way, two items were created *ad-hoc* for the present study (i.e., “I think it is important to ‘like’ or comment on my friends’ posts,” “I think it is important to share photos or videos on social media depicting me and my friends together”). In this sample, the internal consistency was McDonald’s ω = 0.68. Participants rated their level of agreement with each item on a five-point scale (from 1 = not at all, to 5 = completely true). A latent variable was then computed with the two items (factor loadings are reported in Figure 2).

#### 2.2.6 Data analysis

First, zero-order correlations between all study variables were computed (Table 1). After that, to test our hypotheses, a structural equation modeling (SEM) was performed using Mplus 8.3 (Muthén and Muthén, 2017), with the maximum likelihood estimator. In the tested model (Figure 1), perceived friendship quality was the dependent variable. The three perceived social media features (i.e., availability, quantifiability, and visualness), participants’ beliefs on other-oriented use and their perception of friends’ other-oriented use were the independent variables. Adolescents’ self-oriented and other-oriented use were included in the model as mediators. Finally, we controlled for participants’ age. Model fit indices (CFI, RMSEA, and SRMR) and *R^2^* of each endogenous variable were examined to evaluate the goodness of the model. Then we calculated indirect effects by using bias-corrected bootstrap confidence intervals, with 10,000 bootstrapped iterations that were considered to be significant when zero was not included in the 95% confidence interval.

**Table 1:** Descriptive statistics and correlations in the whole sample.

	1	2	3	4	5	6	7	8	9	10	11
1 Validation	_										
2 Intimacy	0.49^**^	_									
3 Companionship	0.45^**^	0.46^**^	_								
4 Availability	0.14^**^	0.20^**^	0.25^**^	_							
5 Quantifiability	0.10^**^	0.12^**^	0.16^**^	0.43^**^	_						
6 Visualness	0.13^**^	0.14^**^	0.13^**^	0.23^**^	0.19^**^	_					
7 Perceived norms about other-oriented use	0.27^**^	0.31^**^	0.31^**^	0.17^**^	0.17^**^	0.22^**^	_				
8 Beliefs on other-oriented use	0.18^**^	0.21^**^	0.15^**^	0.22^*^	0.18^**^	0.34^**^	0.39^**^	_			
9 Self-oriented use	0.14^**^	0.22^**^	0.18^*^	0.11^**^	0.08^*^	0.21^**^	0.46^**^	0.34^**^	_		
10 Other-oriented use	0.16^**^	0.28^**^	0.21^**^	0.18^**^	0.12^**^	0.16^**^	0.40^**^	0.28^**^	0.43^**^	_	
11 Age	−0.02	0.05	−0.01	0.16^**^	0.01	−0.01	−0.06	−0.03	−0.01	−04	_

*N* = 751; ^*^p < 0.05, ^**^p < 0.001.

Initially, the model was tested on the entire sample of adolescents. Subsequently, a multi-group invariance test was performed to examine measurement equivalence across gender groups (males vs. females), as a prerequisite to conducting meaningful between-group comparisons (Vandenberg and Lance, 2000; van de Schoot et al., 2012). To assess whether the assumption of invariance was acceptable, change in value of fit indices (i.e., ΔCFI, ΔRMSEA, and ΔSRMR) was considered. Negligible variations, that is, a ΔCFI smaller than 0.01 and a change smaller than 0.015 in RMSEA and SRMR, were considered indicative of invariance (e.g., Cheung and Rensvold, 2002; Chen, 2007). After this, a multi-group analysis was run to evaluate the model separately for both genders (males vs. females) and the null hypothesis of parameter equalities across groups was tested using the Wald chi-square test in Mplus (Muthén and Muthén, 1998–2015; Wang and Wang, 2012, pp. 276–278).

## 3 Results

### 3.1 Descriptive statistics and correlations

Table 1 shows the descriptive statistics and correlations among the variables analyzed in the study. As expected, significant correlations between perceived social media features, adolescents’ and friends’ online activity and friendship quality emerged.

### 3.2 SEM analysis on the whole sample

Results from the SEM model conducted on the whole sample showed a good fit between the model and the data: χ^2^_(90)_ = 303.802, *p* < 0.001; CFI = 0.936; RMSEA = 0.057, 90% CI [0.050, 0.064], SRMR = 0.038. Figure 2 shows standardized coefficients. As expected, regarding the direct associations, perceived friendship quality was positively associated with perceived group norms (*β* = 0.37, *p* < 0.001), and with participants’ other-oriented use (*β* = 0.10, *p* < 0.05). Further, perceived group norms were positively associated with participants’ both self-oriented (*β* = 0.36, *p* < 0.001) and other-oriented use (*β* = 0.36, *p* < 0.001) and with their beliefs on other-oriented use (*β* = 0.35, *p* < 0.001). Moreover, participants’ beliefs on other-oriented use were positively associated with their self-oriented use (*β* = 0.21, *p* < 0.05); however, contrary to what we expected, it was not significantly associated with the frequency of other-oriented use. Finally, with regard to the three perceived social media features, availability was found to be directly associated with participants’ other-oriented social media use (*β* = 0.15, *p* < 0.05) and with perceived friendship quality (*β* = 0.26, *p* < 0.05). Visualness was significantly linked only to beliefs on other-oriented use (*β* = 0.42, *p* < 0.001), while no significant effects were found between quantifiability and the other considered variables.

Overall, the model explained 29.6% of the variance for perceived friendship quality, and the explained variance for the mediators was 21.4% for other-oriented use, and 28.1% for self-oriented use.

In addition to the direct paths, two significant indirect effects were identified (Table 2). The strongest indirect association was found between visualness and self-oriented use via beliefs about other-oriented use, and a smaller one between perceived group norms and participants’ self-oriented use, via beliefs about other-oriented use. Further, we also found two other indirect associations of borderline statistical significance, between perceived group norms and friendship quality and between availability and friendship quality via participants’ other-oriented use.

**Table 2:** Unstandardized indirect effects.

		Outcome					
Independent variables	Mediators	Friendship quality		Other-oriented use		Self-oriented use	
		ES	CI 95%	ES	CI 95%	ES	CI 95%
Perceived norms about other-oriented use	Other-oriented use	0.019	−0.002 to 0.040	-	-	-	-
Perceived norms about other-oriented use	Self-oriented use	0.000	−0.022 to 0.019	-	-	-	-
Perceived norms about other-oriented use	Beliefs on other-oriented use	0.000	−0.037 to 0.032	0.044	−0.037 to 0.128	0.089	0.023 to 0.162
Perceived norms about other-oriented use	Beliefs on other-oriented use→	0.002	−0.001 to 0.007	-	-	-	-
	Other-oriented use						
Perceived norms about other-oriented use	Beliefs on other-oriented use→	0.000	−0.005 to 0.005	-	-	-	-
	Self-oriented use						
Beliefs on other-oriented use	Other-oriented use	0.006	−0.005 to 0.023	-	-	-	-
Beliefs on other-oriented use	Self-oriented use	0.000	−0.016 to 0.016	-	-	-	-
Availability	Other-oriented use	0.010	−0.002 to 0.027	-	-	-	-
Availability	Self-oriented use	0.000	−0.006 to 0.005	-	-	-	-
Availability	Beliefs on other-oriented use	0.000	−0.017 to 0.012	0.010	−0.024 to 0.058	0.021	−0.031 to 0.079
Availability	Beliefs on other-oriented use→	0.000	−0.001 to 0.003	-	-	-	-
	Other-oriented use						
Availability	Beliefs on other-oriented use→	0.000	−0.002 to 0.002	-	-	-	-
	Self-oriented use						
Quantifiability	Other-oriented use	−0.002	−0.010 to 0.005	-	-	-	-
Quantifiability	Self-oriented use	0.000	−0.004 to 0.005	-	-	-	-
Quantifiability	Beliefs on other-oriented use	0.000	−0.007 to 0.010	−0.001	−0.027 to 0.030	−002	−0.045 to 0.037
Quantifiability	Beliefs on other-oriented use→	0.000	−0.001 to 0.001	-	-	-	-
	Other-oriented use						
Quantifiability	Beliefs on other-oriented use→	0.000	−0.001 to 0.001	-	-	-	-
	Self-oriented use						
Visualness	Other-oriented use	0.001	−0.013 to 0.016	-	-	-	-
Visualness	Self-oriented use	0.000	−0.011 to 0.009	-	-	-	-
Visualness	Beliefs on other-oriented use	0.000	−0.064 to 0.060	0.074	−0.038 to 0.247	0.150	0.042 to 0.281
Visualness	Beliefs on other-oriented use→	0.003	−0.002 to 0.013	-	-	-	-
	Other-oriented use						
Visualness	Beliefs on other-oriented use→	0.000	−0.008 to 0.008	-	-	-	-
	Self-oriented use						

ES, Estimate; 95% CI, Bias-corrected bootstrapped confidence interval.

### 3.3 Test of gender differences

First, configural invariance was tested by running an unconstrained model, that is, all parameters were freely estimated in the two groups. This analysis showed an acceptable fit: χ^2^_(124)_ = 227.767, CFI = 0.961, RMSEA = 0.048, SRMR = 0.039. Then, two subsequent analyses in which parameters were progressively constrained to be equal across groups confirmed metric invariance (ΔCFI = 0.003; ΔRMSEA = 0.000; ΔSRMR = −0.005), and scalar invariance (ΔCFI = 0.014; ΔRMSEA = −0.006; ΔSRMR = −0.004). Full results are reported in Table 3.

**Table 3:** Fit indices for gender invariance tests.

	χ2	df	CFI	ΔCFI	RMSEA	ΔRMSEA	SRMR	ΔSRMR
Configural invariance	227.8	124	0.961	_	0.048	_	0.039	_
Metric invariance	243.5	132	0.958	0.003	0.048	0	0.044	−0.005
Scalar invariance	287.1	140	0.944	0.014	0.054	−0.006	0.048	−0.004

*N* = 751.

Once measurement invariance was confirmed, we compared unstandardized path estimates between gender groups. However, no significant differences between males and females emerged. The overall Wald test of parameter constraints was not significant [Wald *χ^2^*_(25)_ = 23.599, *p* = 0.54], indicating that all paths were equal across groups. Similarly, constraining all paths to be equal did not worsen the fit of the model.

## 4 Discussion

The present study was developed according to the core principles of the Transformation Framework (Nesi et al., 2018a,b) and recent research findings regarding friendship relationships on social media (Yau and Reich, 2018). Based on findings from a previous study (Angelini et al., 2022), this study aimed to overcome its exploratory design and to further investigate the positive links emerged between adolescents’ perception of some of the characteristics of social media (i.e., availability, visualness, and quantifiability) and specific components of friendship quality (i.e., validation, intimacy, and companionship). Specifically, in this study, we “zoomed” into specific activities on social media (i.e., active social media use) through which adolescents and their friends behave within their relationships (e.g., chatting, posting content, “liking” or commenting on friends’ posts); further, we took into account the potential role of perceived group norms about active social media use in contributing to explain perceived friendship quality. Overall, the results confirmed that different perceptions of social media features are–both directly and indirectly–linked to perceived quality of friendship and that peer influence processes are active also in the social media context; that is, perceiving specific social media features is associated with the way adolescents and their friends use social media to interact with each other and, in turn, with positive friendship experiences.

### 4.1 Direct associations of other-oriented social media use on friendship quality

Regarding our first hypothesis, as expected, results of the SEM analysis showed that both participants’ other-oriented use and their perceived group norms about it were positively associated with friendship quality. That is, the more adolescents use social media to interact with and react to friends’ content (e.g., by liking or commenting on their posted material) and perceive that their friends enact similar behaviors on social media, the greater the level of validation, intimacy, and companionship they experience in their friendship relationship. Frequent and quick contacts with peers through social media, in fact, can support friends’ interactions in different and new ways (Nesi et al., 2018a; Angelini et al., 2022). For example, adolescents report being more satisfied with their friendship-related experiences when they use social media to interact with their friends and with the content they have posted (i.e., in an other-oriented way). Thereby, other-oriented use provides occasions for supportiveness among friends, thus creating more intimacy; moreover, adolescents experience greater companionship and feel like friends are always with them, thanks to the immediacy and availability of online interactions. In addition, the possibility to send or receive daily positive feedback (e.g., in terms of “likes,” comments or visual content) is associated with feelings of reciprocate validation within the relations with friends. Finally, these online experiences are also associated with reciprocal feelings of acceptance and approval and, consequently, connectedness with friends. Furthermore, the reciprocal other-oriented use has an important function for relationship maintenance. Please note that our findings should be interpreted in light of the cross-sectional design, which does not allow us to determine the direction of observed associations. In fact, another plausible explanation is that other-oriented social media use and perceived friendship quality are both outcomes of other underlying individuals’ variables, such as personality traits. For example, higher sociability can drive both the preference for specific social media activities (e.g., other oriented use) and perceived friendship quality; that is, adolescents with good social skills provide their friends with positive feedback both within face-to-face interactions and when they interact on social media, thus perceiving higher friendship quality.

In this regard, a three-wave longitudinal study with a sample of Dutch adolescents (Angelini et al., in preparation), showed a bidirectional association between different patterns of social media use during adolescence and the perceived quality of close relationships, indicating that activities on social media both reflected and were driven by real-life friendships. Specifically, adolescents with poor relationships with friends were also less active on social media, and, in turn, perceived a lower quality of relationships over time.

As the current study did not control for specific personality traits or offline experiences with friends, future longitudinal research should account for these characteristics in order draw causal conclusions with greater certainty.

Moreover, given the reciprocity that characterizes friendship relationships, we could expect that both friends’ and participants’ other-oriented use would have been associated with friendship quality with the same strength. In contrast, greater friendship quality was more strongly associated with perceived norms about friends’ other-oriented use, compared to participants’ other-oriented use. Reciprocity is a central feature in friendship experiences and refers to friends’ mutual engagement in the relationship (Parker and Asher, 1993). Previous studies indicated that reciprocal relations with friends positively impact youth well-being, such as feeling less lonely and better liked by peers (see, e.g., Parker and Asher, 1993; Salmivalli and Isaacs, 2005). In this sense, the stronger association of perceived group norms with friendship quality, compared to participants’ other-oriented use, might indicate that, when reporting the level of their friendship’s quality, participants tend to attribute more importance to the perceived friends’ online behavior, that is, to feel more satisfied when they perceive that their friends are committed to the relation. Further understanding of how perceived friends’ online activity, also comparing different types of friends’ social media use in terms of both strategies and content, is associated with adolescents’ satisfaction with their friendship relationships is an important venue for future research.

### 4.2 The role of group norms on adolescents’ active social media use

A further aim of the present study was to examine peer (friend) influence processes on adolescents’ active social media use and beliefs about it. Specifically, we found that perceived group norms about other-oriented use was directly and positively associated with both participants’ beliefs about using social media in an other-oriented way and their active social media use. As within a process of mutual reinforcement, the more adolescents perceive that their friends use social media to frequently like, comment on, and interact with their own posted content, the greater importance they tend to attribute to this behavior and the more they use social media in an active way. With regard to the latter association, we expected that perceived norms would have been more strongly associated with participants’ other-oriented use, than self-oriented use, since the two variables measured similar behaviors by participants and their friends. However, perceived group norms were found to be associated with both types of active social media use (self-oriented and other-oriented use), with the same strength. The perception that friends use social media to frequently respond to and interact with one’s own online content, indeed, could work as a descriptive social norm and give value to this behavior, thus motivating adolescents to also use social media in an other-oriented way. This finding is consistent with the literature about between-friends similarity (e.g., Mazur and Richards, 2011; Marino et al., 2020) indicating that the more adolescents perceive their friends’ behavior to be frequent, the more they are likely to behave in this way themselves. At the same time, however, higher perceived norms about other-oriented use may make adolescents expect that friends will react and interact with their shared posts on social media, thereby increasing the frequency to which they post new content. To note that subjective feedback relevance and engaging in active social media use may also be affected by individual differences in terms of self-esteem or perceived social status in the peer group (Diefenbach and Anders, 2022).

Additionally, our findings unexpectedly showed that participants’ beliefs about other-oriented use were not associated with the frequency of their other-oriented use; however, it was linked with the frequency of self-oriented use. This result may be due to the association between perceived norms and participants’ other-oriented use, and, in addition, could be explained by the fact that adolescents seem to attribute importance to the use of social media in an other-oriented way, but they enact it only when they perceive that their friends do. Similarly to what we have discussed so far, along with the direct paths, perceived norms were also found to be indirectly associated with self-oriented use via beliefs on other-oriented use. In sum, we believe that these results support the hypothesis that social norms within the friendship network play an important role in adolescents’ choice of how to behave in the social media context and, in turn, on perceived friendship quality. Such findings are in line with what discussed within the Transformation Framework about how social media can alter or amplify certain already heightened developmental tasks during adolescence, such as social reward sensitivity. As such, adolescents’ tendency to engage in specific online activities relies on motivations related to socially rewarding mechanisms (Nesi and Prinstein, 2018; Nesi et al., 2018a,b). However, these findings should be interpreted through the lens of participants’ self-reported perceptions of how their friends behave on social media. In fact, there may be potential differences in the way adolescents perceive their friends’ behaviors on social media and what their friends actually do. Research that compares participants’ self-reports with objective data derived from their social media activities, such as the actual number of likes or comments from friends, would contribute to a clearer understanding of whether certain experiences and behaviors are over- or underestimated. Consequently, understanding the dynamics between adolescents’ perceptions and their actual social media experiences could help identify more vulnerable young people, as these individuals may tend to interpret their online experiences in ways that reinforce their expectations. This research could provide valuable insights into the psychological mechanisms behind the formation and maintenance of social norms in online environments.

### 4.3 The contribution of social media context

Regarding our third hypothesis, different social media features were found to be linked to participants’ other-oriented use and to beliefs on it and, as well to perceived friendship quality. More in detail, perceived availability was found to be positively associated both with friendship quality and adolescents’ other-oriented use. That is, adolescents who perceive that the context of social media offer the opportunity to reach out their friends without time or location restrictions are more likely to engage in other-oriented social media use as well as to experience greater satisfaction in their relationships with friends. The availability of social media, indeed, facilitate adolescents in communicating and interacting with friends, also those living geographically distant, whenever they would like to do so. This increases opportunities for adolescents to show affection to friends, to offer support, or to have fun with them by “liking” by commenting on their posts, and thereby experience a higher satisfaction in the relationship (blinded for review; Nesi et al., 2018a,b; Yau and Reich, 2018; Angelini et al., 2022).

Additionally, perceived visualness was positively related to participants’ beliefs about other-oriented use. That is, the perceived emphasis on visual material (e.g., photographs, videos, and alike) in the context of social media leads adolescents to attach more importance to the use of social media in an other-oriented way. The characteristic of visualness, indeed, offers adolescents the opportunity to interact with friends through new forms of communication that may shape their opinions about how important it is to “like” or comment on friends’ last video, or to share a photo depicting them together on a special occasion. To note that, while the three features may be perceived by all adolescents when they are active on social media, the level of salience and perceived presence of each feature may differ depending on individual characteristics and on which platform is used to interact with friends.

In addition, with regard to indirect paths, visualness was found to be associated with self-oriented use via beliefs on other-oriented use. That is, the more adolescents perceive the value of using multimedia communication on social media, the more they think it is important to react to and interact with posts (e.g., photos or videos) shared by friends and, in turn, the more they tend to use social media in a self-oriented way. Thus, similarly to what we discussed before, adolescents’ expectation of their friends’ online feedback lead them to frequently access their own social media to post new visual content.

Regarding the perceived characteristic of quantifiability, no significant association was found in our sample. Of course, lack of significant effects may have a variety of explanations. This model was conceptually based on the findings of a previous study in which quantifiability was found to be significantly associated with perceived validation among friends; moreover, in the present study, the bivariate correlations suggested that perceived quantifiability of social media is associated with the other considered variables. The absence of statistically significant paths in the overall model indicates that availability and visualness play a more relevant role, compared to quantifiability, in determining adolescents’ use of social media to interact with their friends. In other terms, active social media use, beliefs about it and friendship quality are more clearly related to adolescents’ perception of the possibility of connecting with friends regardless of the time or place and to take advantage of visual communication allowed by social media, than by the possibility of literally counting social indicators (e.g., number of comments, likes, and friends). Further research is needed to better investigate this possible interpretation.

### 4.4 Differences across gender groups

Another aim of the current study was to examine potential differences across gender groups. However, results did not show significant differences between the two groups. That is, the social media context, as well as the relation between social norms and the frequency of active social media use among adolescents appear to be similarly associated with perceived friendship quality, for both males and females. Despite existing differences in the ways males and females use social media (e.g., females generally spend a greater amount of time and are more active online), perceived friendship quality benefits from other-oriented social media use consistently across gender groups. These results may have important implications, particularly concerning the need to enhance males’ awareness of the potential benefits of increased other-oriented social media activities for relational well-being. However, research on gender differences in the association of perceived friendship quality with social media experiences is still limited. Further studies should aim to replicate this research, and test more specific hypotheses about gender differences.

### 4.5 Limitations

Some limitations of the current study should be acknowledged. First, the cross-sectional design does not enable us to infer the causality or direction of the observed associations, and thus to rule out a possible reverse causality (Rohrer et al., 2022). For example, as discussed above, it could also be that perceived friendship quality explains the way adolescents use and perceive social media, or that individual characteristics (e.g., sociability) drive the way they use social media to interact with friends and, in turn, their perceived friendship quality. Moreover, as discussed for gender differences, given the positive association of other-oriented use with friendship quality, we also believe that our findings support the idea that being more active on social media (in terms of interactions with friends online) represents an opportunity to improve one’s social skills, as well as positive experiences with friends and, in turn, perceived friendship quality. Future investigations should aim to include specific control variables and try to replicate these findings using a longitudinal design and involving larger and cross-cultural samples.

Second, self-report questionnaires have inherent shortfalls (particularly in measuring automatic behaviors such as smartphone usage). Also, to note that the lack of adequate instruments for measuring the frequency of specific online behaviors meant that we could measure these concepts with only one or two items, mostly created *ad-hoc*. With regard to the perceived social media features scale we needed to use a newly developed measure and, although we found preliminary evidence of its factorial structure (Angelini et al., 2022), further analyses are needed to fully establish its validity and reliability. Future research should try to reduce potential measurement error by increasing the number of items and also include objective measures (e.g., asking participants to install an application that keeps track of their specific activities on social media) to overcome some limits of self-reports. Further, we asked participants to assess their friends’ social media use. While the perception of how friends’ use social media is certainly important for how adolescents themselves act online and its use is common practice in this field of research, to better investigate the reciprocal nature of friendship relationships during adolescence it would also be interesting to replicate this study with a sample of dyad of friends. This would allow to perform more detailed dyadic analyses. In addition, while we tested the indirect paths via the two dimensions of active social media use (i.e., self-oriented use and other-oriented use) and we focused on specific perceived social media features (i.e., availability, quantifiability, and visualness), there are certainly other online behaviors (e.g., online status seeking) and contextual dimensions (e.g., algorithm on social media) that may be important to investigate. Finally, beyond the positive dimensions of friendship (validation, intimacy, and companionship) it would also be interesting to study the negative side of friendship relations, that is, to understand which perceived social media features may be linked with online behaviors that can lead to perceive lower friendship quality in adolescence (e.g., higher conflict or online victimization experiences among peers).

### 4.6 Implications

Despite these limitations, potential implications for preventive and educational programs with adolescents can be highlighted. Schools and educators could be informed about these results in order to find support for the implementation of specific interventions aimed at promoting a healthy use of these platforms. For example, group discussions focused on perceived group norms and peer communication would be useful to share expectations about social media use, and specific online activities may facilitate positive experiences with friends, both offline and online. Thereby, adolescents can turn to peers to make meaning of their personal and shared experiences. Further, to raise adolescents’ awareness of the contextual functioning of social media, it can be useful to discuss their perception of the social media features (e.g., availability and visualness) and how they are associated to their activities on social media within adolescent groups. Most social media, in fact, are designed to facilitate social connection, and adolescents should be made aware of this potential in order to reap the relational benefits of their use.

## 5 Conclusion

In conclusion, this study contributes to the expanding body of literature about adolescents’ online behavior and friendship relationships on the social media. Specifically, our findings suggest that friends who engage more in other-oriented use may be more satisfied with their friendship relationships. In addition, these results show the relative importance of peer influence processes on adolescents’ active social media use and beliefs on it. Our findings support the notion that the social media context is important in the study of relational functioning and well-being within peer relationships among contemporary adolescents, and confirm that the novel behavior (e.g., commenting, liking photos), which characterize interactions with friends, may support the psychological and relational purposes of friendships in the offline environment. Social media provide youths with new opportunities to disclose affective states (e.g., via posted content) and to foster social interactions with friends (via other-oriented use), as a way to maintain and strengthen relational and emotional developmental tasks during adolescence.

## Author’s note

The data reported in the manuscript were collected as part of a larger project about adolescents’ social media use. However, there is no overlap between these data and the data reported in any previous study.

## Data availability statement

The raw data supporting the conclusions of this article will be made available by the authors, without undue reservation.

## Ethics statement

The studies involving humans were approved by Ethics Committee for Psychological Research of the University of Padova. The studies were conducted in accordance with the local legislation and institutional requirements. Written informed consent for participation in this study was provided by the participants’ legal guardians/next of kin.

## Author contributions

FA: conceptualization, methodology, data curation, formal analysis, and writing—original draft. GG: conceptualization, methodology, supervision, and writing—review and editing. CM: supervision, writing—review and editing. RE: writing—review and editing. All authors contributed to the article and approved the submitted version.
